# Digital twin-driven variant design of a 3C electronic product assembly line

**DOI:** 10.1038/s41598-022-07894-x

**Published:** 2022-03-09

**Authors:** Douxi Yan, Weinan Sha, Dewen Wang, Jiafeng Yang, Shenghui Zhang

**Affiliations:** 1 State Key Laboratory of Precision Electronic Manufacturing Technology and Equipment, Guangdong University of Technology, Guangzhou, 510006 Guangdong China; 2 Guangdong Provincial Key Laboratory of Computer Integrated Manufacturing System, Guangdong University of Technology, Guangzhou, 510006 Guangdong China

**Keywords:** Mechanical engineering

## Abstract

Large-scale personalization is becoming a reality. To ensure market competitiveness and economic benefits, enterprises require rapid response capability and flexible manufacturing operations. However, variant design and production line reconfiguration are complicated because it involves the commissioning, replacement, and adaptive integration of equipment and remodification of control systems. Herein, a digital twin-driven production line variant design is presented. As a new technology, the digital twin can realize the parallel control from the physical world to the digital world and accelerate the design process of the production line through a virtual–real linkage. Simultaneously, the actual production line can be simulated to verify the rationality of the design scheme and avoid cost wastage. Four key technologies are described in detail, and a production line variant design platform based on digital twin is built to support rapid production line variant design. Finally, experiments using a smartphone assembly line as an example are performed; the results demonstrate that the proposed method can realize production line variant design and increase production efficiency.

## Introduction

In the current market environment, the challenge for manufacturers is to produce products with a short lifecycle and a high degree of customization within a short duration^1,2^. With continuous improvement in the level of economic and social development, the variant design of the production line aims to meet the various needs of customers. Based on the design object, variant designing adopts technologies and methods such as selection, configuration, and variant to form a new design scheme and fulfill the production-level requirements of the enterprise to strive for stability, efficiency, low consumption, and high quality of the production process. Currently, serial design is used as the manufacturing system design process, which is developed and designed according to the sequence of the whole line scheme, intermediate equipment, control system, execution system, integration test, installation, and commissioning. However, serial design cannot consider the overall situation of the manufacturing system and involves few connections and integration parts that are highly dependent on the experience of designers, which will inevitably result in the creation of many unreasonable designs. Variant design of production lines needs to consider not only the static configuration of the workshop, such as the capacity, equipment selection, layout, and constraints of the legacy systems, but also the dynamic operation process, including key factors such as the motion rhythm design, capacity balance, product processing form, and workshop logistics. The production line design also extends to the integration of equipment, control systems, and control software, which involves the adaptive modification of the execution system and engine and the iterative optimization of the execution efficiency. The static resource allocation scheme needs to verify the dynamic execution effect, and the dynamic execution effect needs to be improved through the adjustment of the static configuration scheme. Therefore, the process of workshop design is highly complex, and the research and development cycle is long.

Recently, the mobile phone consumer market has witnessed rapid development and diversified and personalized consumer demands for mobile phones have been increasing. In production workshops, high-frequency production changes have been commonly observed in the 3C electronics industry, including mobile phones^3^. High levels of integration and rapid replacement are typical attributes of 3C electronic products. In addition, we can observe traits such as increasing personalization demand, high mobile phone upgrade frequency, multiple product specifications, urgent delivery time, frequent occurrence of the single insertion phenomena, and frequent requirement of changing the production line of enterprises, which includes replacing equipment, switching processes, configuring production parameters, adjusting production plans, and implementing differentiated orders to fulfill customer demands. The variant design method, which is based on reusing previous design resources, fulfills the consumer demands in a short time by ensuring cost and quality. It can effectively improve the current product design with respect to complexity, diversity, and time consumption. It is important to effectively reuse previous design knowledge for the successful implementation of the variant design. Restricted by the traditional design concept of the de-standardization and customization of automation equipment, the standardization, modularization, and flexibility of automation equipment have not been established in the mobile phone assembly and production automation industries. Simultaneously, many automation enterprises are still in the development and manufacturing stage of nonstandard automation equipment. This challenges the design paradigm and production solutions, resulting in slow production line design, affecting production efficiency, and directly reducing the competitiveness of enterprises and economic benefits. The traditional variant design method requires the manufacturer to stop production, replace related equipment, and adjust system parameters. However, owing to the lack of advance verification of the transformation scheme, the production line design after the transformation is unreasonable, affecting the actual production activities and resulting in low production capacity.

As an essential application of smart manufacturing, it describes the operation of a system from the perspectives of virtual and reality^4^ and effectively supports the customized design, rapid reconstruction, and distributed integration of production lines. In particular, the digital twin maps the physical world to the virtual world, realizing real-time mapping, interaction, integration, and data fusion between the physical and virtual systems and truly simulating the action and workflow of the physical equipment in the process of the production line^5^. Simulations can be carried out based on the digital world to support various production and manufacturing in the physical world. By determining the communication protocol standard, instruction format standard, field information format, etc., a digital twin network is constructed to realize the interconnection and intercommunication between the digital world and the physical world and finally realize parallel control. Based on the digital twin, complex manufacturing processes can be integrated to achieve closed-loop optimization of the production line design, commissioning, verification, integration, and service. It realizes data interconnection between the digital and physical worlds, providing more accurate services for the product design process.

The rest of this study is organized as follows. A literature review on the variant design method and digital twin technology is provided in “Related works” section. “Variant design framework” section describes the architecture of the variant design method. “Key technologies” section introduces and discusses four key enabling technologies, including hierarchical modularization, virtual commissioning, reuse of design knowledge, and extensible distributed communication framework. “Demonstrative prototype and case study” section presents illustrative applications and discussions to verify the proposed model. Finally, conclusions are presented in “Conclusion” section.

## Related works

Variant design refers to the extraction of an existing design scheme or design plan based on specific modifications to develop a product with a design similar to that of the original. Generally, it does not destroy the basic principles and basic structural characteristics of the original product; instead, it is a type of fusion-based parameter change or partial structural adjustment performed to realize fast, high-quality, and low-cost design^6–8^. The variant design of products drives functional change through structural change in an agile manner. The change achieved via the variant design begins from the user domain and spreads to the structural domain, functional domain, and use process domain of products. Product family planning, modular parametric variant design^9^, knowledge-based variant design^10^, variant design based on product assembly model^11^, case-based variant design^12^, and other design methods have emerged in combination with product assembly structure and characteristic product parameters. The key to product variant design is to establish a multidomain and multi-use model of product variant structure and define the evolution process^13^. Yang et al.^14^ proposed a rapid modeling method for product skeleton and a parametric design method for products and established an assembly model and product skeleton model template of a series of products based on the similarity principle. Wang et al.^15^ also studied the application of CBD technology in product variant design and established a basic analytical model of variant design. Through practical application, the deformation design principle and method can reduce design intensity and shorten design time. Compared with the change caused by product variant design, the change caused by the production line is more diversified. In particular, the alteration of users and products triggers a change in the production line structure configuration, equipment action, work-in-process (WIP) movement, control network, manufacturing execution system, and execution engine. Liu et al.^2^ proposed a digital twin-drive rapid design method for automated flow shop design and developed a double-layer iterative coordination mechanism to achieve optimal design performance of functions required by automated flow shop systems. Further, for the design of a process-based intelligent manufacturing system, the CMCO (configuration design-motion planning-control-development-optimization-decoupling) design architecture was proposed, the iterative logic of CMCO design model was elaborated, and the prototype manufacturing system design platform based on digital twin brother was developed^16^. Variant design mainly focuses on the variant design of products. The key to the variant design of products is to establish a multidomain and multi-use model of product variation structure and define the evolution process. Compared with the change effect of product variant design, the change effect of the production line is more diversified, including the change from users and products, the change of production line structure configuration, equipment action, WIP movement, control network, manufacturing execution system. The variant design of production lines is usually studied around a specific topic, such as production line balance^17–19^, equipment configuration optimization^20–22^, and layout design^23,24^. However, the design of the production line must be characterized, reasoned, and decided through the global design idea^25^. In practice, the variant design of the production line is complex and high-dimensional, not only including configuration design at the static level but also logic design at the dynamic level. Therefore, describing the system from all directions and dimensions and establishing relationships between design dimensions is necessary to achieve a fast, agile and accurate design.

Digital twin technology is increasingly being applied in various fields. Many scholars have performed in-depth research and practice on this technology and have reported extensive research results^5,26,27^. It has powerful simulation capability and can support design tasks or validate system attributes^28^. Digital twin technology can also effectively realize the fusion and management of multisource heterogeneous dynamic data throughout an entire product lifecycle along with the optimization and decision-making of various product development and production activities. Tao et al.^29^ proposed a product design framework based on digital twin and demonstrated the effectiveness of the proposed method through an example. Digital twin describes system operation from the virtual and real perspectives and can provide robust support to the customized design, rapid reconstruction, distributed integration, transparent monitoring, virtual operation, and maintenance of workshop production lines. It also opens up a new route for manufacturing system modeling and simulation analysis based on trial-and-error, reducible, near-physical, high-fidelity, and virtual–real synchronous verification and highly interactive iterative optimization. Guo et al.^30^ proposed a modular method to help construct a digital twin considering the frequent changes in the design stage of a factory. Designers can then evaluate design schemes based on the digital twin and quickly identify design defects, thus saving time. Leng et al.^31^ proposed a new digital twin-driven joint optimization method for the design and optimization of large automated high-rise warehouses. Periodic optimal decisions can be obtained by establishing a joint optimization model and then fed back to the semi-physical simulation engine in the digital twin system to verify the results. Yan et al.^32^ also proposed a rapid custom design method for a novel furniture panel production line based on a digital twin. This method has the characteristics of interactive virtual reality mapping and fusion. It can provide design guidance and decision support services at the design stage, generate engineering analysis to solve coupling problems, and ultimately generate authoritative design solutions for manufacturing systems. Yi et al.^33^ also proposed a digital twin reference model for intelligent assembly process design together with a three-layer intelligent assembly application framework based on a digital twin. The working mechanism of assembly process planning, simulation, prediction, and control management in the virtual space layer was discussed in detail. Leng et al.^4^ proposed a digital twin manufacturing network physical system for the parallel control of intelligent shops in a large-scale personalized mode. Through the physical connection of a distributed digital twin model, all types of manufacturing resources can be integrated to form dynamic autonomous systems and jointly create personalized products.

Many studies have shown that digital twin technologies have the characteristics of a design task and verification system. Herein, considering a mobile phone assembly line as an example, a production line variant design method driven by the digital twin technology is proposed. The modeling and simulation capabilities of the digital twin enable rapid variant design and solution validation of the production line, reducing variant design time and research costs.

## Variant design framework

**Figure 1:**
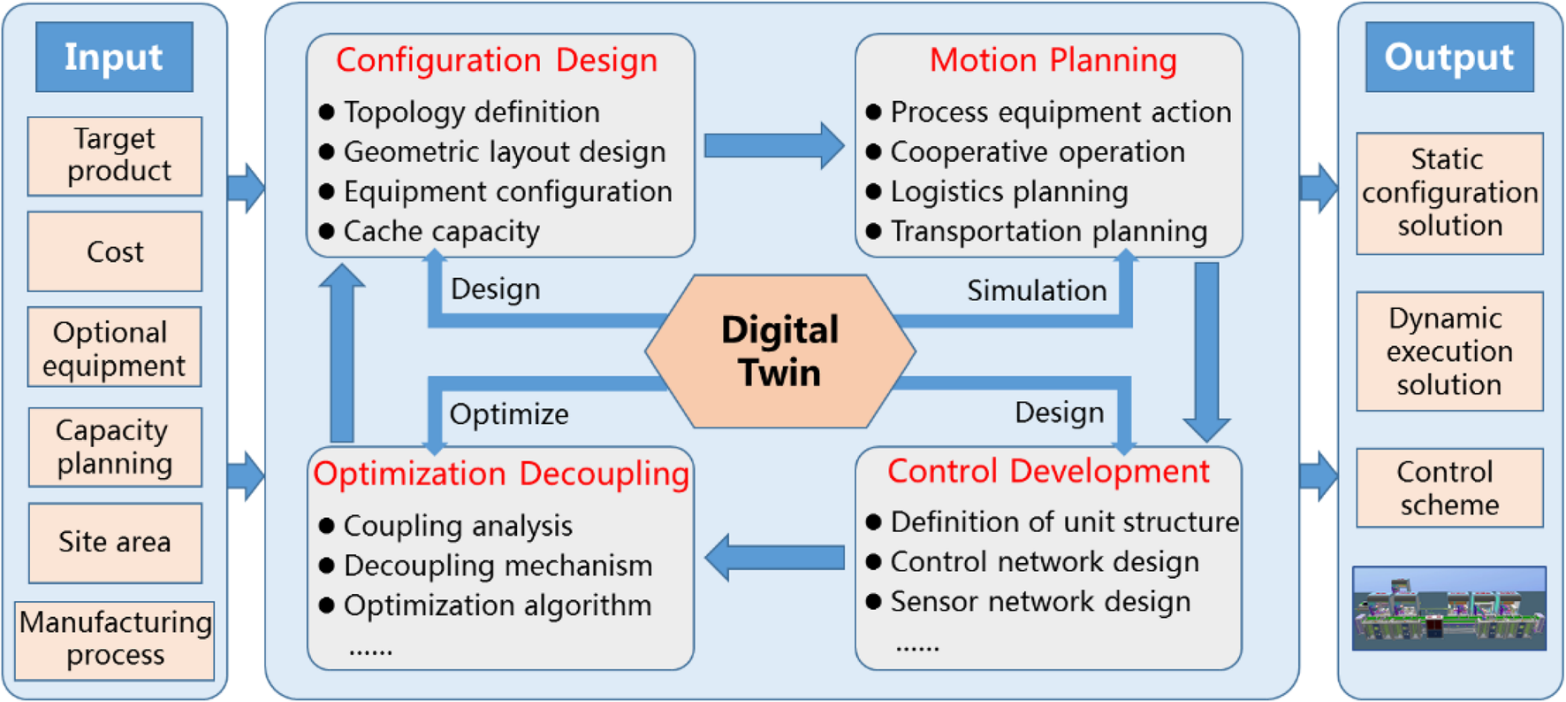
Variant design framework.

In previous literature^16^, the design of intelligent systems has been described as involving four stages: configuration design, motion planning, control planning, and optimization decoupling. On the basis of the definitions of these four stages, we can obtain a good understanding regarding the entire process of manufacturing system design. In accordance with previous literature and the personalization requirement prevalent in the market, this study proposes a closed-loop design logic for a four-type linkage driven by the digital twin technology. As shown in Fig. 1, the design input includes the target product, capacity, site area, budget, manufacturing process, and other personalization requirements. The process is conducted in a configuration design–motion, planning–control, development–optimization, decoupling closed-loop, and parameter correlation between various stages. All of these lead to corresponding changes and the associated parameter changes in other design stages. Configuration design is a static configuration process that mainly includes the definition of the production line topology and equipment parameter configuration. Motion planning, control development, and optimization decoupling all belong to a dynamic simulation and execution optimization process. The coupling problem is implemented step by step according to the static configuration and motion commissioning of equipment, unit control logic verification, and production line optimization. The coupling problem is formed through production planning, resource scheduling, and order grouping and batching in the smartphone assembly line. An analysis and solution mechanism of the coupling problem structure is key to the optimal operation of the whole production line, forming the engine of its dynamic operation. The optimization operation of the execution engine causes iterative changes in the design process, and the parameters of each design stage change until the engine obtains the final static configuration scheme and dynamic execution scheme. The convergence of the closed-loop iterative process is considerably important to the design process and ensures the accuracy and validity of the design results. The design outputs include the static configuration and dynamic execution results as well as the control and layout schemes. The measurement indices of production line variant design include equipment selection, line layout, cost control, and capacity requirements. Simultaneously, it is important to consider whether the scheme fulfills the requirements of differentiated production and performs adaptive adjustment as well as optimization.

## Key technologies

### Hierarchical modularization of equipment

Time-consuming and challenging production-line design links are achieved through the different specifications of 3C manufacturing equipment, numerous accessories, a wide range of processing equipment customization, multiple integration interfaces, a long research and development cycle, complex and rapid integration, and high research and development costs. A modular design method is used to design the manufacturing equipment. Four interface types are defined: the communication, software, mechanical, and electrical interfaces. The purpose of the method is to improve the adaptability and interchangeability of the equipment, and the modularity of the equipment is crucial for fulfilling personalized needs. Standardized communication interfaces facilitate communication among different equipment and solve equipment isolation caused by inconsistent communication among equipment. The software interface defines the application programming interface (API) software specifications, provides the program interfaces, and defines the protocol content to facilitate the connection and integration among the heterogeneous software systems. The primary function of the mechanical interface is to transfer energy and motion and solve the matching between mechanical and electrical systems, including the power transmission and change of motion direction. Further, the electrical interface defines the power supply and ventilation mode of the equipment. Different processing equipment can be quickly energized and ventilated using standardized electrical interfaces.

Based on the encapsulation of the four interface types and according to the division of equipment function, we propose a hierarchical modular method for 3C equipment. Under the mechanical platform generalization–electrical layout standardization–auxiliary body configuration–actuator customization–control system regarding the secondary development system deconstruction path and the standardized characteristics of the extraction module, we form a standardized interface between the different modules, a definition of transportation, a processing subsystem of the plug-and-play structure, and an electrical and software bus. We formed a series of available platforms and standard interfaces, realizing the standardization of the subsystem interface, including the transmission, handling, and processing subsystem.

**Figure 2:**
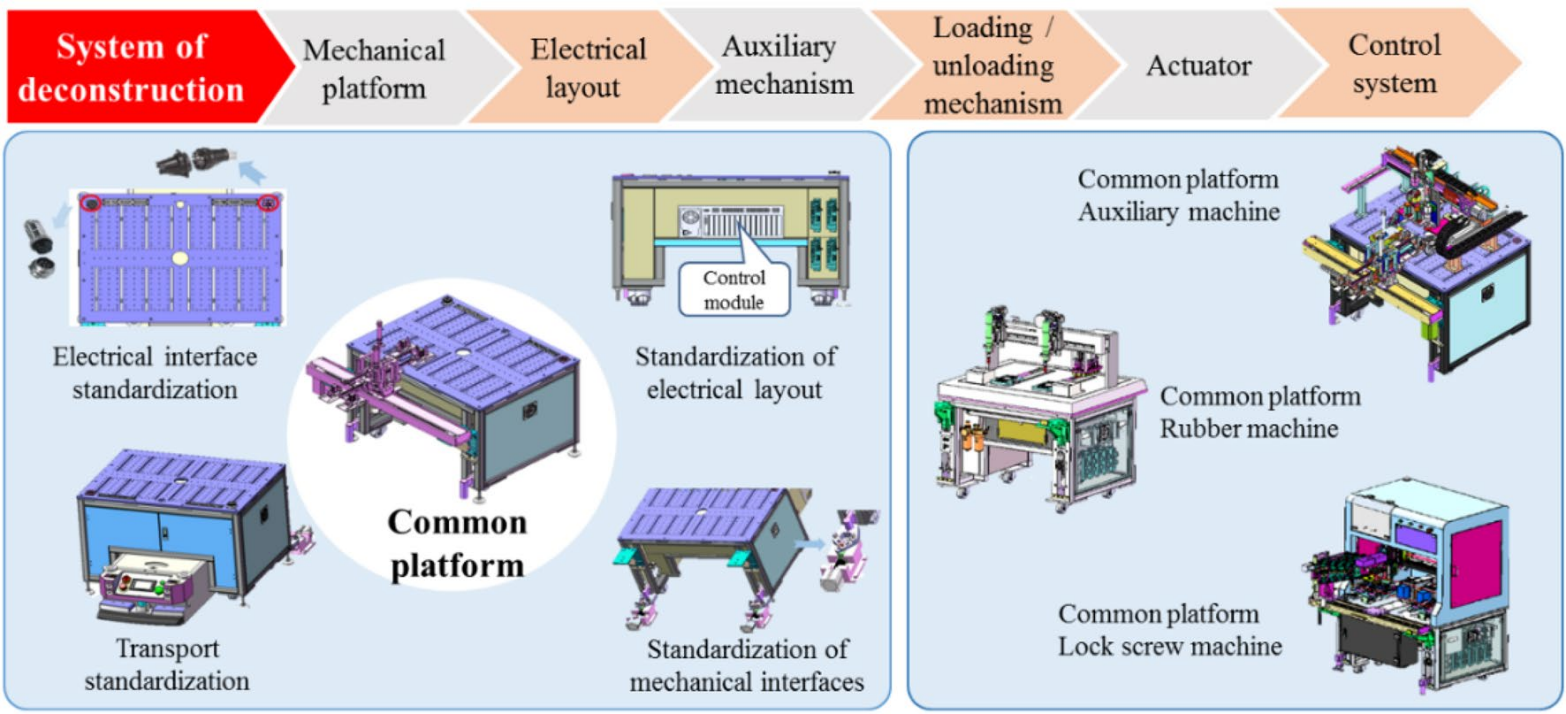
Hierarchical modularization of equipment.

Through these four standardized interfaces, the reconfiguration and reusability of the manufacturing equipment are improved, and the plug-and-play function of the equipment in the production line can be realized. As shown in Fig. 2, considering an automatic smartphone assembly line as the object, by defining the basic automation component architecture and general platform library through the design template, the automatic equipment of the "general base + manipulator + customized module" was formed by combining the loading and unloading manipulators and other customized execution modules.

### Digital twin-driven virtual commissioning of equipment

The existing development model of automatic production lines and equipment is a serial method ranging from mechanical and electronic control design to software verification. Using the serial development method, design problems at all levels are gradually exposed in the integration commissioning stage. System-level problems can occur, affecting the functional integrity of the automation equipment, accuracy of the control logic, and performance reliability, which can result in a production line performance system that does not fulfill production needs. Twin technology based on digital virtual equipment commissioning can run without affecting the production line for virtual equipment under the condition of commissioning and test equipment control logic. Its action is by design requirements, which can be determined in the early stages of the equipment control scheme design to avoid faults. To accelerate the construction of the production-line process, control program errors caused by the time costs can also be determined.

**Figure 3:**
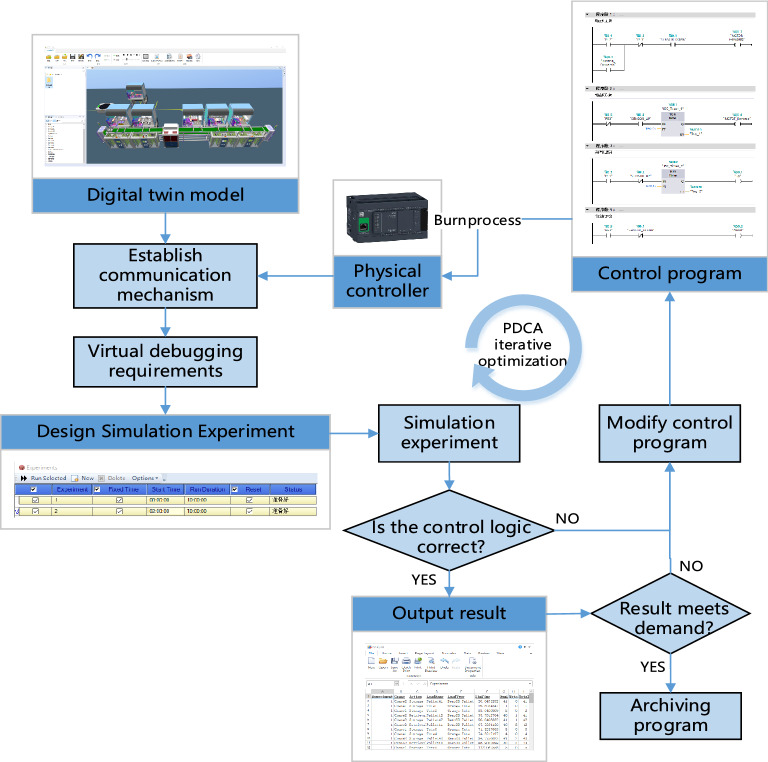
Digital twin-driven virtual commissioning of equipment.

Figure 3 shows the virtual commissioning process of the equipment based on the digital twin technologies. First, we selected the PLC controller and communication protocol, determined the input and output equipment and point address, and designed the control program. Then, according to the movement mode and logistics form of WIP, the execution position and process parameters of the equipment were determined, and the control scheme was configured. Further, the simulation experiment was performed based on the actual production situation. The input signal (analog output signal) of the PLC controller was simulated in the digital twin, and the signal was transmitted to the hardware PLC controller through network port wiring. The control program then fed the output signal (analog input signal) back to the digital twin platform to realize closed-loop control commissioning. Additional simulation experiments were performed to verify whether the control logic was correct, to output results, and to analyze whether it fulfills the requirements; or else, the PDCA process (planning, execution, inspection, and action) was executed circularly, and the control procedure was optimized iteratively.

After the PDCA cycle was iterated for the control logic of equipment, the optimized program was archived and burned into the physical equipment controller, and the commissioning, iteration, optimization, and verification of the control logic of all equipment throughout the entire line were finally completed. The management and control system issued instructions to drive the digital twin model, simulating the process control of order placing. The server was responsible for receiving sensor signals from equipment and distributing them to each equipment controller via industrial Ethernet. The equipment controller outputs the execution signal after receiving the sensing signal according to the program logic, uploads it to the server, and forwards it to the digital twin execution model action control. Then, it implemented semi-physical commissioning as well as debugged and verified the process control logic of the control system again.

### Code-level design knowledge reuse

**Figure 4:**
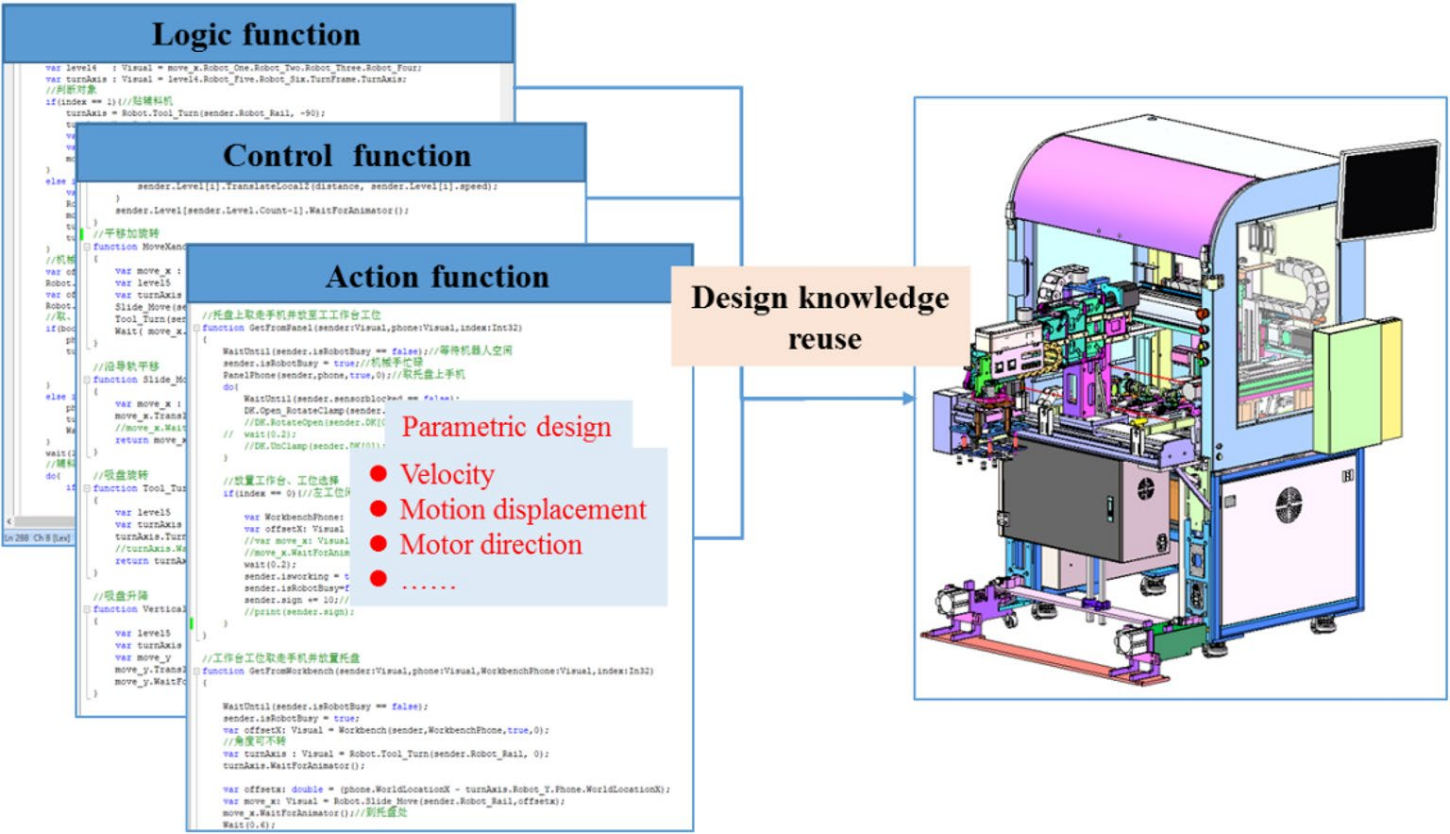
Code-level design knowledge reuse.

Reuse and effective dissemination of design knowledge is the basis of the rapid production line design, and fully exploring the fragmented, unstructured, and tacit knowledge related to equipment use is necessary. The primary form of WIP motion is generally basic motions (linear, flip, rotation, or space curve motion) or compound motion (turning, lifting, stacking, sorting, arrangement, loading, and unloading motions). Basic and compound motions are further combined to form another compound form of motion (series, merging, and crossing motions). The design of the equipment and WIP movement are important parts of the variant design for production lines. The action function was encapsulated to form a parameterized action function, which was classified and sorted to form an equipment action knowledge base. Clustering analysis technology was then used to summarize its internal rules, and a clustering knowledge base that can be searched quickly and in order was established to promote the standardization of fragmented design knowledge. A carrier representation of fragmented knowledge is more conducive to the spread of design knowledge and can be used as a reference by designers to achieve effective reuse of design knowledge. As shown in Fig. 4, the functions are summarized as follows.

#### Action function

Motion code encapsulation encapsulates the most specific motion of each motion mechanism, and each motion is encapsulated into a separate function. For example, the left–right linear motion of the manipulator along the Z-axis direction, up and down linear motion along the Y-axis direction, and loose clamp motion of the manipulator end fixture can be encapsulated as three motion functions, respectively. The input parameters of the motion function are usually related to the mechanism motion, including its speed, stroke, and direction.

#### Control function

The control function needs to be encapsulated according to function granularity, from fine to coarse. Fine-grained control function encapsulation is achieved by calling the code encapsulation of the movement method and control mechanism for a specific function of the machine. Coarse-grained control function encapsulation is achieved by calling the fine-grained control function encapsulation method for the general equipment function. The control function encapsulation of input parameters is usually related to equipment functions, including the selection of objects and function mode.

#### Logic function

The logic function encapsulates the entire running logic of the equipment, and the response process of the equipment under a certain input is encapsulated separately into a method separately. Logic function encapsulation can also be divided into virtual operation mode and digital twin synchronous-mode encapsulation. In virtual operation mode, the input encapsulated by logical functions usually determines the other objects used in the simulation scenario on the equipment. In the digital twin synchronous mode, the input encapsulated by the logic function is typically used to monitor the change in the attributes of the corresponding PLC points.

### Extensible distributed communication framework

**Figure 5:**
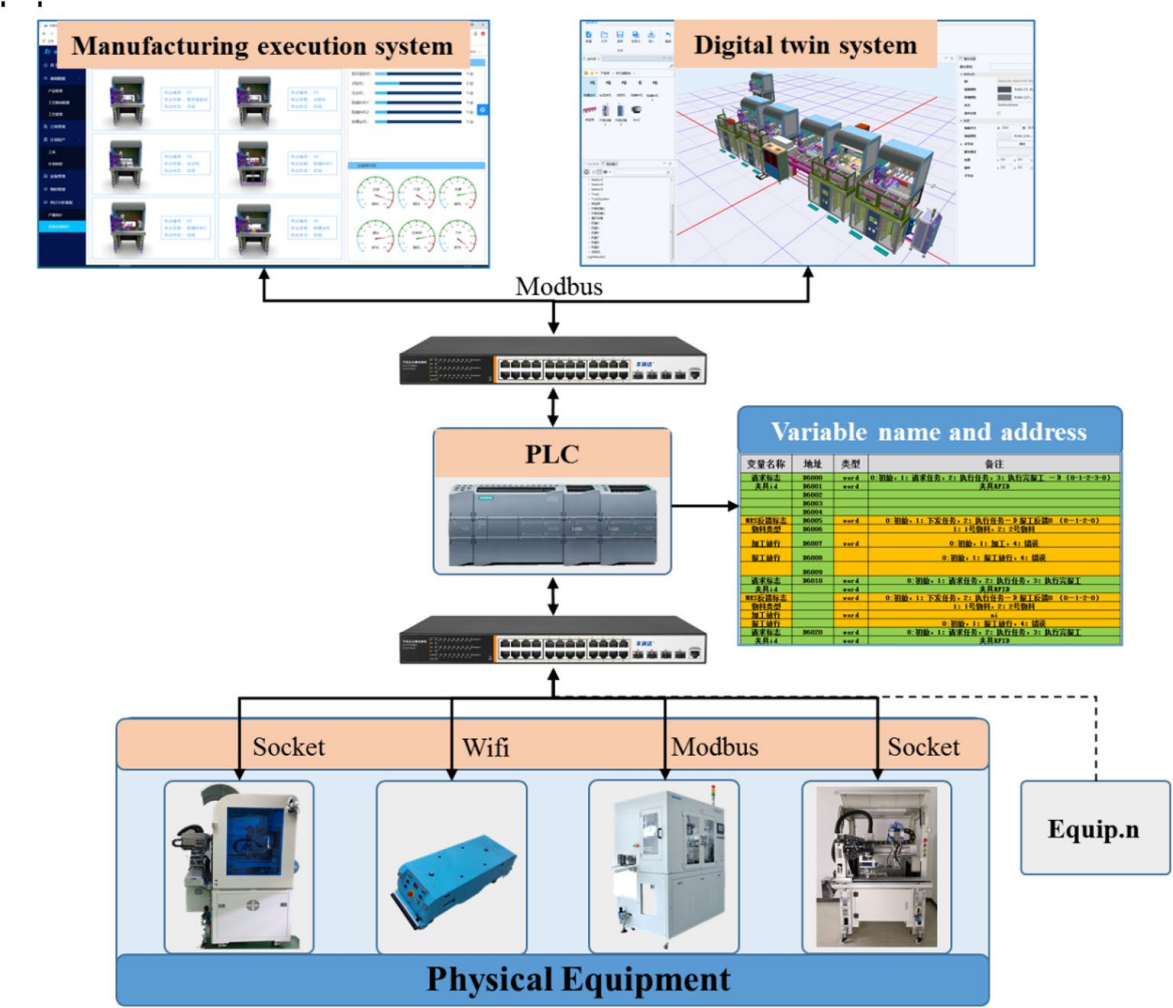
An extensible distributed communication architecture based on Ethernet.

The variant design of production lines includes the adjustment and replacement of hardware equipment and the alteration of software, such as the adjustment of the control program and network. The centralized control structure of a production line generally adopts a top-down, fixed, hierarchical structure. With this rigid architecture, adjusting the configuration to form new functions is complex, and maintenance and reconfiguration costs are high when introducing recent changes, such as adding or replacing machines^34^. Constructing a novel control network in a short period of time using a production line variant design is difficult. This study proposes a scalable distributed communication architecture based on Ethernet. The communication framework is shown in Fig. 5. With the distributed communication architecture adopting Ethernet as a connection mode, all equipment is connected through the Ethernet, and each manufacturing unit can communicate with the other units through network configuration.

The names of the control variables and input/output (I/O) point addresses are defined on the PLC. Different physical equipment is assigned separate I/O addresses to achieve distributed control of a single equipment. In the communication architecture, we integrated various communication protocols, including Modbus and Socket, to overcome the obstacles between the digital twin model and heterogeneous physical equipment, such as robots, software systems, and scanners.

A point on the physical PLC is bound with a point on the soft PLC of the digital twin system to realize the virtual–real linkage, which is critical in learning the digital twin. The virtual data in the digital twin system communicates with the physical equipment in real-time through the protocol and based on the binding and mapping relationship between the soft and physical PLC addresses, the digital model drives the physical equipment to move, or the physical equipment drives the digital model to transfer by varying control variables. The manufacturing execution system circularly scans the alteration of the point position on the PLC to monitor the movement status of the equipment in real-time.

## Demonstrative prototype and case study

### Digital twin system prototype

**Figure 6:**
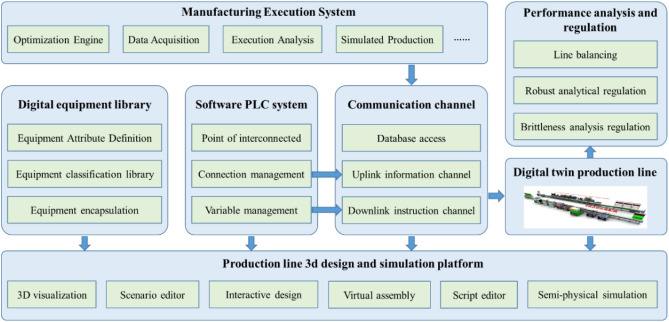
Digital twin-driven production line deformation design platform.

Based on the proposed enabling technology, a digital twin-driven variant production line design platform was constructed. The platform architecture is shown in Fig. 6. In the production line design system, a digital equipment library was formed based on design experience and design knowledge, the equipment was classified and encapsulated, and the related interfaces and attributes were parameterized. The purpose of this process was to realize rapid production line variant design and reduce the redundancy of the design action. The soft PLC system was designed, which makes it possible to recognize the digital twins. The point position information of the PLC was mapped to the software, and the communication channel was further built, including the uplink information channel and the downlink instruction channel. These were the basis for realizing the virtual and real synchronization of the digital model and physical equipment. The platform also included a performance analysis and control module, which analyzes and controls the robustness and brittleness of the production line. The manufacturing execution system (MES) acts as the execution engine for the entire platform to place production orders and perform simulation analyses of the design platform. The MES analyzes the simulation results in real-time and adjusts and optimizes the variant design scheme based on the results.

### Design of a smartphone assembly line based on digital twin

**Figure 7:**
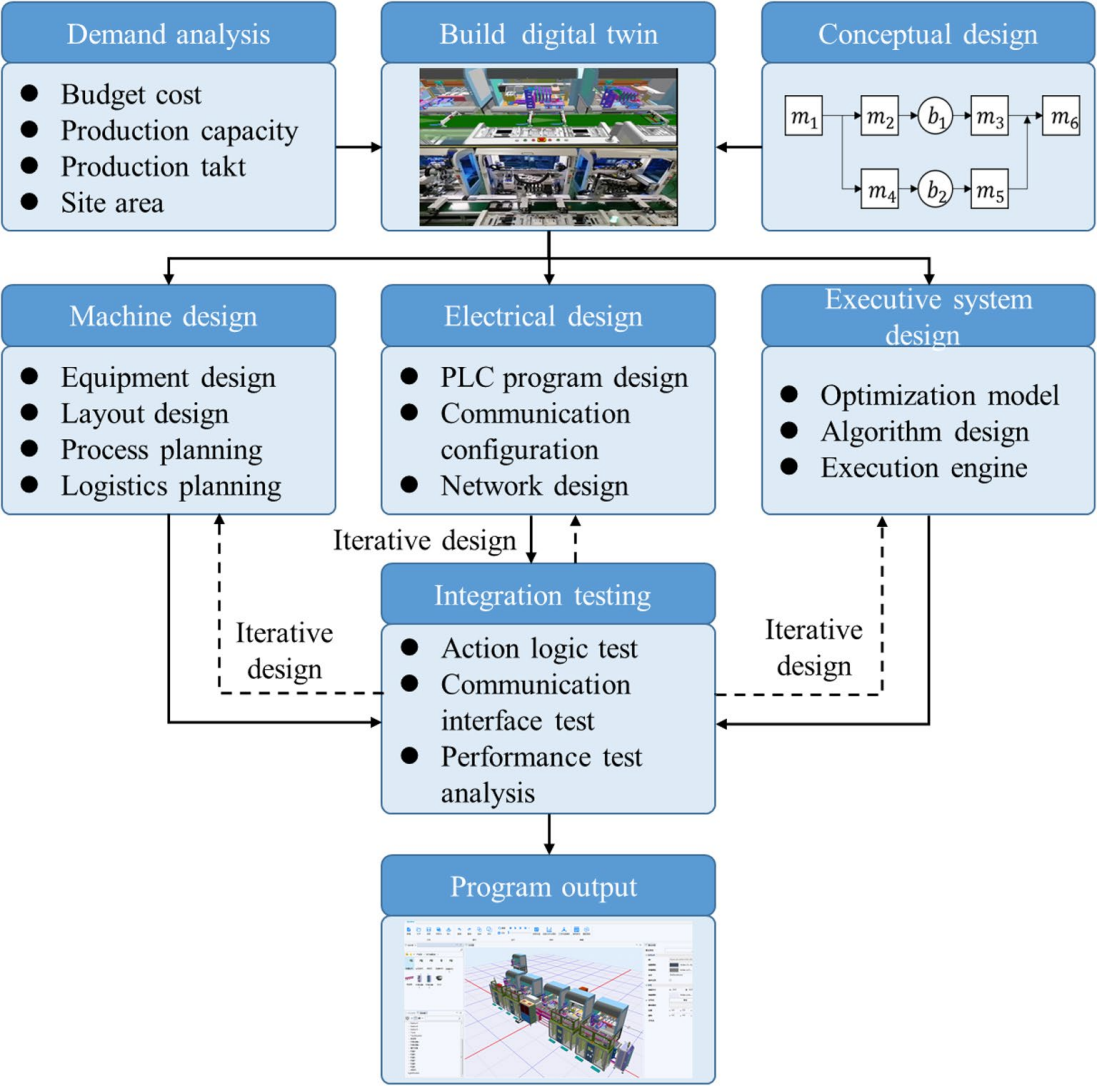
Production line variant design steps.

**Figure 8:**
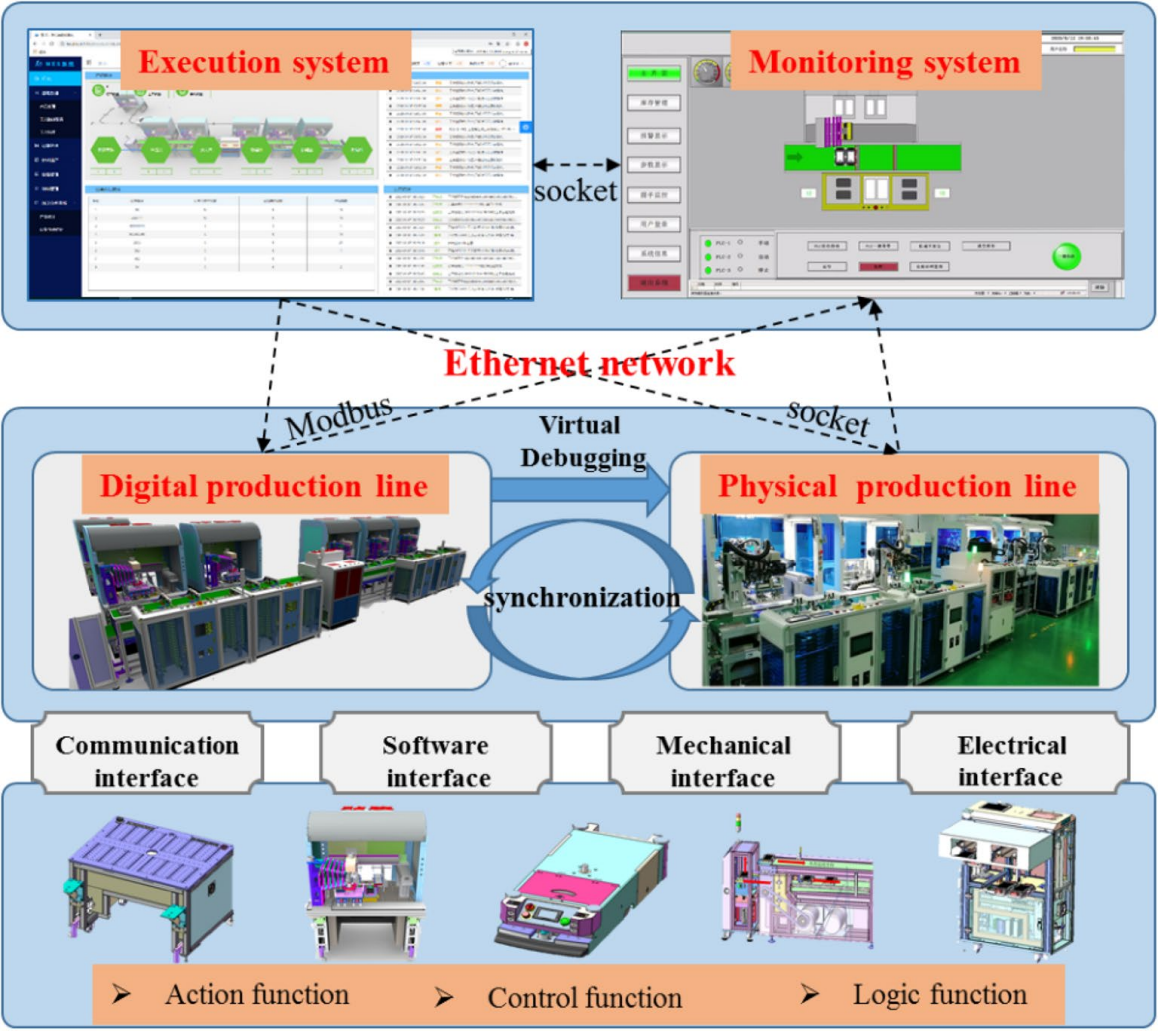
Smartphone assembly line.

Based on the aforementioned key technologies and platforms, a variant design platform for a 3C electronics production line was built according to the design steps shown in Fig. 7. As shown in Fig. 8, a typical smartphone assembly line process was selected to indicate the construction of a digital dual smartphone assembly line. The operation of the mobile phone assembly line included dispensing, sticking TP double-sided adhesive, TP pressing, sticking accessories, and locking screws. The process and equipment of the smartphone assembly line adopted the design method of the “standard machine + manipulator + customized processing module.” The standard machine is conducive to the use of AGV replacement equipment. The software, electrical, mechanical, and network interfaces were encapsulated to realize the plug-and-play function, saving the operation time of workers replacing lines and meeting the actual time requirements of factory replacing lines. Some of the design features are listed below.

- *Middle reflux design* Mobile phone assembly is based on three main components: the screen, middle plate, and back shell. In the assembly process, the screen and the middle pallet are assembled first, followed by the middle pallet and back shell. The production line logistic scheme of the middle section turnover–tray backflow was designed to avoid manual pallet handling.
- *Standardized open equipment platform* Typical process equipment is designed with open architecture, and the mechanical bus adopts a universal equipment base plate. Installation holes and positioning slots with good universality were arranged according to the characteristics of 3C industry equipment, realizing the direct positioning and installation of common mechanical structural parts or motion modules.
- *Flexible loading and unloading mechanism design* The frequent replacement of processed products can cause changes in material and type. An adaptive tension mechanism (tensioning wheel to control the friction force) was developed. The feeder mechanism could adapt to the automatic stripping of over 85% of electronic accessories. The motor power and start and stop controls of the feeder mechanism were connected to the hardware of the equipment control system by the connector, which can realize the hot-swap and advance refueling of the stripping feeder on the equipment.

### Comparative analysis

We compared the mobile phone assembly line without digital twin technology with the mobile phone assembly line designed using digital twin technology and selected key indices to evaluate the performance of the assembly line. Takt time represents the time it takes to produce each item. Makespan refers to the maximum processing time for a product. UPPH is the labor capacity per unit time which is the ratio of the number of tasks to the number of hours worked times the number of workers. Equipment utilization is the ratio of total uptime to total available time. Production line balance refers to the average of all production processes and the adjustment of workload, which is proportional to the total working time of each process and the number of workers multiplied by the bottleneck working time. Table 1 summarizes the comparison results, according to which the UPPH value was found to have been significantly increased, doubling the number of techniques previously used. The makespan also exhibited a decrease of 83.5% due to the open architecture of the production line, which used telescopic manipulators to grab WIP and speed WIP flow. After implementing the digital twin method, the production line could run in the virtual platform of the digital twin according to the actual demands. To adjust and balance the use rate of the equipment, a bottleneck position of the production line was effectively avoided, and the use rate of the equipment was effectively improved by 129.8%, and line balance increased by 25.5%.

**Table 1:** Comparison between the conventional and improved production lines.

Items	Original	DT-variant design	Improvement (%)	Decrement (%)
Takt time (s)	11	6	–	45
Makespan (s)	681.2	112.6	–	83.5
UPPH (U/P/H)	13	27	107.7	–
Equipment utilization (%)	34.2	78.6	129.8	–
Line balance (%)	72.5	91	25.5	–

## Conclusion

This study introduces a method of variant design for production lines based on digital twin. Further, a code-level design knowledge reuse method and an extensible distributed communication architecture were proposed. A production line variant design platform based on a digital twin was built based on the proposed enabling technology to support rapid production line variant design. The successful variant design of an automatic mobile phone assembly line demonstrated that the proposed variant design method based on digital twin could provide intelligent simulation as well as a simulation verification function for design. The production line variation method proposed in this study can guide the production line design and replacement of 3C electronic enterprises. For the implementer of the digital twin technology in shop floor manufacturing, the proposed method is reproducible and can reduce the design change time of the production line, as well as realize the verification and online testing of the design scheme, which can bring real economic benefits to the enterprise. However, many issues remain to be resolved. First, Physical equipment and digital twin model use different controllers to control the motion, which will lead to the adoption of asynchronous synchronization and communication delay. Second, the fault interference in the production process will affect the accuracy of the design scheme; therefore, learning to model the disturbance factors and clarify the influence on the design is very important. Third, the digital twin model is highly significant to accumulating and reusing design knowledge. In the process of the production line design, the effective reuse of design knowledge and design linkage is the key to improving the rapidity and accuracy of customized design of the whole line. Summarizing the design experience and design cases and constructing a new knowledge design method can help accelerate the variant design of the production line and shorten the design cycle.
